# The acceptability of evidence-informed guidance for parents in talking to their children about weight

**DOI:** 10.1186/s12889-023-16267-6

**Published:** 2023-07-14

**Authors:** Fran Baber, Fiona B Gillison, Elisabeth B Grey

**Affiliations:** 1 Centre for Motivation and Health Behaviour Change, Department for Health, University of Bath, Bath, UK; 2 Population Health Sciences, Bristol Medical School, University of Bristol, Bristol, UK

**Keywords:** Parent-child communication, Health communication, Parenting, Child wellbeing, Child weight

## Abstract

**Background:**

Many parents express concern about the impact of talking to children about weight on their self-esteem and wellbeing. The aim of this study was to explore the perceived relevance, utility and acceptability of new guidance for parents on talking to children about weight, developed to apply theory, evidence and expert advice into practice.

**Methods:**

For this qualitative study, parents and public health practitioners (PHPs) were recruited from ten local authorities in England, through the National Child Measurement Programme between June and September 2021. Participants were sent a copy of the guidance document and took part in an interview approximately one week later. Interviews were transcribed verbatim and explored using thematic analysis.

**Results:**

12 parents and 15 PHPs took part, and were similar in their responses reporting the guidance to be acceptable, relevant and helpful. Theme 1 explored how the guidance reduced perceptions of stigma and blame through the perspective and tone that was adopted. Theme 2 explored how the guidance could provide reassurance and increase confidence as a result of case study examples, and specific tips and advice. Theme 3 explored the extent to which participants perceived the advice to be realistic and how it could fit with existing PHP practice. Suggestions for improvement included adapting for relevance for lower income families and providing separate advice for parents of older and younger children.

**Conclusions:**

The guidance was perceived as relevant and needed; it showed potential to reduce parents’ negative affect and concerns, and improve confidence around talking to children about weight.

**Supplementary Information:**

The online version contains supplementary material available at 10.1186/s12889-023-16267-6.

## Background

Childhood obesity remains a significant global public health concern [1]. In England, one in ten children have obesity when they start primary school (age 4–5 years), rising to one in five by the time they leave in year six (age 10–11 years) [2]. Obesity in childhood is likely to track into adulthood [3], and obesity in adulthood is associated with increased risk of chronic illness [4]. Children who have overweight or obesity may also experience stigma and bullying because of their weight, which is associated with low self-esteem, social withdrawal and mental health problems [5].

In England, obesity prevalence is monitored through the National Child Measurement Programme (NCMP) delivered by Local Authorities (LAs), which aims to measure the height and weight of all children in Reception and Year 6 attending primary schools [6]. Although parents can choose to withdraw their child from the programme, annual participation rates are high, at around 95% before the COVID-19 pandemic [7]. Following NCMP measurement, most LAs notify parents if their child is above or below a healthy weight, and offer advice and support [8]. The aim of providing this information is to enable parents to make informed decisions around the care of their child, given the influence that parents have on the diet and physical activity of children of primary school age [9, 10]. However, research suggests that this feedback does not usually prompt parents to make changes to their children’s weight-related behaviours [11, 12]. While some parents simply dismiss or disagree with weight feedback [13–16], other parents may react more negatively, reporting distress, shock, disappointment, anger [17], or fear that labelling children as overweight can contribute to the development of eating disorders and poorer wellbeing [6, 14]. A systematic review exploring parents’ experiences of being told their child is overweight suggested that negative reactions also reflect parents’ lack of self-efficacy and confidence in addressing their child’s weight [13], and in particular their lack of confidence in talking to their children about their weight without harming their child’s wellbeing [18].

A systematic review of studies exploring how different types of parental weight-talk could influence children’s mental health and wellbeing showed that some forms of parent-child communication, such as criticising children’s weight and encouraging dieting, are indeed associated with poorer wellbeing and mental health outcomes [19]. Similarly, a large longitudinal study has reported an association between parents recognition of their child’s overweight status, and poorer mental health outcomes in children [20]. However, other studies have shown that constructive parental conversations that focus on promoting health behaviours, such as healthy eating and physical exercise, and avoid direct reference to weight loss can have a beneficial effect on wellbeing [19, 21, 22]. Taken together, this evidence suggests that raising parental awareness of their child’s weight status may not be a neutral activity, and that ensuring parents are appropriately guided towards the responses that are positive for their children is important. Guidance already exists for healthcare professionals on how best to talk to parents when their child is identified as having overweight [23]. However, until recently there has been no equivalent guidance available to support parents in talking to their children.

The present study forms part of a larger project which aimed to develop a usable guidance tool for parents, underpinned by evidence-based best practice and relevant theoretical techniques. Development of this guidance involved (i) collating and reviewing existing evidence and theory, including systematic reviews of the literature [24]; (ii) engaging with stakeholders (including parents, children and public health practitioners (PHPs)); and (iii) an iterative Delphi process to create and refine draft versions. The full process is reported elsewhere [25]. The present study aimed to explore the perceived relevance, utility and acceptability of this new draft guidance with parents and PHPs, including school nurses and other practitioners involved in child health screening. We included PHPs as key stakeholders, as they are essential for acceptance, support and implementation of the final guidance resource. Low parental response rates in previous studies demonstrate challenges in engaging parents to participate in research about childhood weight, particularly parents of children with overweight or obesity, and those from lower socioeconomic status and minority ethnic groups [11, 12, 14]. Therefore, given anticipated challenges in engaging parents, including PHPs also added their broader experience and perspectives of working with families from more diverse backgrounds.

## Methods

### Design

This was a qualitative interview study. Ethical approval was granted by the Research Ethics Approval Committee for Health at the University of Bath (reference number EP 20/21 039).

### Sample and recruitment

Eligible participants were (a) parents of children in reception (aged 4–5 years) or Year 6 (aged 10–11 years) who had been measured as having overweight or obesity in the NCMP in the 2020/2021 school year; or (b) PHPs who had direct contact with families through their role in delivering the NCMP. Recruitment and data collection took place remotely via Teams or telephone between June and September 2021. Throughout this article, we use the word ‘parents’ to mean both parents and other primary caregivers, such as legal guardians or grand-parents.

To recruit, an advert was distributed to a mailing list of all Local Authority (LA) NCMP leads in England, inviting the LA to take part by distributing invitations to eligible staff and parents. Staff were recruited through internal email and eligible parents through study invitations sent either with or after NCMP results. Information sheets sent with study invitations provided details on the purpose of the study, what taking part would involve and how to contact the research team. We asked LAs to invite every parent on their records who had been informed that their child had been classified as overweight or very overweight, and to encourage participants from lower socio-economic groups a £10 voucher was offered for participation. Although the guidance is relevant to parents of children of all body weights, only parents of children identified as having overweight or obesity were recruited, partly as these families are the primary focus of public health intervention, and partly as most LAs do not provide NCMP results to parents of healthy weight children. Using a gatekeeper in this way allowed us to target the eligible parent and PHP population while avoiding the need for the research team to access the confidential contact details of either study population. However, as each LA operated in different ways, we were not able to standardise the approach to recruitment across LAs or obtain comprehensive details of response rates. We aimed to conduct 10 to 20 interviews with each participant group, stopping recruitment when the dataset was considered adequate for exploration of both parents’ and PHPs’ perceptions [26]. For both groups, recruitment was stopped when the dataset was determined to encompass a range of rich responses and no new ideas were being generated.

### Procedure

Study invitations directed people to an online participant information sheet, which provided the researchers’ names, impetus of the study, and what the findings would be useful for (including a Masters dissertation). This sheet also contained a link to an online survey. Here, volunteers were asked to confirm they met the eligibility criteria, then confirm their consent to participate and provide their preferred contact details. After confirming eligibility and consent, the researchers contacted participants to arrange an interview and send an electronic or paper copy of the guidance, depending on participants’ preference. Interviews were scheduled for approximately one week after receiving the guidance, allowing participants time to read and reflect on it. Interviews were conducted by FB or EG. FB was a female postgraduate health psychology master’s student at the time and underwent training in interviewing technique; she was supervised throughout data collection and analysis by EG, a female postdoctoral researcher, experienced in qualitative interviewing research and with a background in health psychology. Both researchers explicitly encouraged participants to provide positive or negative feedback, explaining that their aim was to make the resource as helpful as possible for parents.

Interview schedules were developed by the research team, informed by literature and our experience of talking to parents about children’s weight. Different schedules were used for parents and PHPs (Additional files 1 and 2). Parent interviews explored: (a) family circumstances and previous experience of talking to their children about weight; (b) parents’ perceptions and comments on each section of the guidance; and (c) how engaging and useful they found the guidance and how/when they would prefer to receive it. The parent interview schedule was piloted with one parent to sense-check the questions and initial acceptability before finalising; no amendments were deemed necessary. PHP interviews explored: (a) their experience in working with parents of children with overweight/obesity; (b) their current practice in terms of providing healthy lifestyle advice and discussing weight; (c) their perceptions and comments on each section of the guidance and whether the content aligned with their practice and their perceived needs of parents; and (d) how the resource could be best implemented. All participants received a £10 gift voucher to thank them for taking part in the study.

### The guidance

The guidance document was in PDF format and titled *‘Talking to your child about weight’* (Additional file 3). Although anticipated to be most relevant to parents of children with overweight or obesity, the guidance is written inclusively for parents of children aged four to eleven of all bodyweights. It comprises six core sections addressing: [1] whether or not to talk to a child about their weight [2], encouraging a whole-family approach [3], advice for how and when to talk about weight [4], promoting positive body image [5], encouragement for parents struggling with their own weight, and [6] example phrasing for responding to common scenarios. Each section contains boxes with text using person-first language [27] and positive, diverse images of children and families enjoying healthy eating and physical activity. Throughout the guidance, boxes titled ‘what could I say…?’ provide examples of conversations starters and responses.

The guidance was developed with input from a broad set of stakeholders to help translate the previous research into information accessible and relevant to parents. The stakeholder group included parents, school nurses, health care professionals, public health specialists as well as academics, and representatives of the Office for Health Improvement and Disparities, all of whom reviewed the various iterations of the guidance, paying particular attention to the readability for parents of all education levels. Full details are reported elsewhere [25].

The content of the guidance drew on health communication theories, relevant literature and the authors’ original research. The framing of the content and messaging aimed to (a) challenge and reduce stigma and stereotypes of childhood obesity, and (b) minimise negative psychological reactance from parents (i.e., the triggering of negative affective states which may result in underestimation or rejection of health messages). To reduce perceived stigma, the determinants of obesity were framed broadly, in order to acknowledge environmental and external factors and discourage perceptions of individual blame [28–30]. Through presenting case of other children and families affected, the guidance also aimed to normalise parents finding out their child is not a healthy weight and struggling with how to respond.

To make the messages more accessible to parents with lower educational levels, and reduce emotional responses to feedback, the guidance incorporates narrative messages [28] in the form of three short case studies informed by narrative communication theories [31]. Narrative messaging can positively influence attitudes towards health behaviours by reducing the likelihood of resistance and dismissal of persuasive health messages [31, 32]. The case studies modelled parents’ responses to weight feedback and/or questions from their children, recognising the challenges of addressing weight whilst exemplifying ways to encourage positive responses and conversations where appropriate. The case studies were based on previous research with parents looking back on their own response to feedback that a child was overweight [33]. They were piloted to confirm they were engaging, successful in presenting novel perspectives, and that parents were easily able to identify with the characters in the case studies [34].

### Data analysis

All interviews were audio recorded, transcribed intelligent verbatim and imported into NVivo version 12. Thematic analysis [35] was conducted to identify patterns within and across data about participants’ views, perspectives and experiences [36]. Following familiarisation through reading and re-reading transcripts, initial thoughts, interpretations and potential codes from the first few interviews were discussed among the research team to develop a coding schedule. This was then applied to the remaining transcripts by FB in a flexible, data-driven manner that allowed for new codes to be added or codes to be collapsed/reorganised. The coding schedule was regularly discussed among the research team throughout the analysis to challenge interpretations and improve credibility of the analysis [37]. All transcripts from parents and PHPs were coded individually, then codes and coded data from both groups were examined together to identify patterns which were salient to the research objectives. These initial patterns informed development of potential themes and subthemes to capture shared perspectives across the dataset. Candidate themes were continually compared with original data, discussed between the research team, and revised and refined to accurately reflect the priorities raised by both groups.

## Results

### Summary of interviews

Participants were 15 PHPs (two male, 13 female) and 12 parents (11 mothers, one father) from 10 LA areas primarily in North East, North West, East and South East England. All the LAs served high proportions of families on low incomes. After completing the online survey and being sent a copy of the guidance, two people did not proceed to arrange an interview, resulting in a 7% drop-out rate at this stage. Interviews ranged from 41 min to 1 h 42 min in length. One interview involved one researcher and two parents (of the same child). Roles of PHP participants included nine school nurses, four child health screening practitioners, one practice development lead and one health improvement specialist. Twelve interviews were conducted via telephone and 15 were conducted via videocall. Where interview extracts are shown, ‘[…]’ denotes unrelated material. Respondents have been assigned numerical identifiers from either the parent or PHP group (e.g., ‘Parent 1’ or ‘PHP 2’).

Parents and PHPs unanimously thought that guidance on talking to children about weight is important, needed and would be helpful. The themes below explore both the context of family weight communication and specific perceptions of the guidance document, including how it fits in this context. There was considerable consistency in feedback from parents and PHPs, so responses from both groups are combined within themes, with clarifications provided where views differed or were only expressed by one group.

### Theme 1: impact on parents’ cognitions and emotions

#### Reducing avoidance, providing reassurance

Many parents reported that they actively avoid talking to their children about weight due to the stigma they perceived to surround childhood overweight and obesity. However, they also recognised that this avoidance could contribute to how children feel about their weight.

*“There’s a bit of a shame or a stigma attached to it if it’s something you’re not talking about, whereas if it’s something in the open it’s more accepted and it’s less shameful for the child.” [Parent 2]*

The guidance was perceived to address this concern, by promoting positivity and kindness, and “*normalising”* weight conversations.

*“I think it’s done in a very positive way. It doesn’t blame anyone in particular, it avoids the kind of negative language like ‘fat’ and anything else that comes along with that. So yeah, to me it’s a very positive document, it encourages change.” [Parent 2]*

*“The language it’s using all the way through is trying to get a sense of normalising discussions about weight, that’s what it gives to me.” [PHP 4]*

Both parents and PHPs framed reasons for avoiding weight discussions as an attempt to protect children, stemming from fears that talking to children about weight could cause eating disorders or damage their self-esteem. Many parents reported lacking confidence in how to handle conversations with their children to avoid these risks. Some PHPs also acknowledged that they lacked confidence in how to directly answer parents’ questions about such risks, or provide advice on what they ‘should’ do, which caused them to “*skirt around the subject”*.

*“There’s so much in social media around kind of eating problems and eating disorders and parents worry that that’s something that they’re going to push that child into if they have those conversations, rather than it probably being the very opposite.” [PHP 12]*

While the guidance did not advocate that parents should talk with their children about weight, it aimed to highlight that there may be times when it would be helpful to do so and that this could be done in a supportive manner. This seemed to provide useful reassurance to parents that talking about weight need not be as risky as they had thought.

*“You just don’t know what the right thing to say is, which is why it’s great to have [the guidance], and you feel like yeah I’m saying the right things now and I’m not going to damage my child.” [Parent 2]*

The case study ‘Mark’s Story’ seemed to be particularly powerful. This was written from the perspective of a child who *wanted* to talk about his weight with his parents, as he was being teased at school and thought his parents could help him. This prompted parents to think differently because it provided a novel child-centred perspective on children’s awareness of their weight.

*“It just suddenly prompted this conversation that I’d been so reluctant to have with her because I’d been frightened […] but actually this made me think about the fact that actually she might be thinking about this already, or this might have been something that’s going on in her mind. So actually to have a bit of courage and push forward a little bit in having that conversation with her.” [Parent 11]*

*“Mark’s story, I think that gave a different perspective. That made me think about how the child may be thinking about it themselves and that hasn’t been picked up on. They may be using this as – waiting for this as an opportunity. I’d never really thought of it like that.” [PHP 11]*

More broadly, the quotes from children interspersed throughout the guidance document were also identified as effective in prompting reflection on how children may feel about their weight and being measured. PHPs suggested that children’s voices were lacking in other parental resources which would make the guidance more novel and engaging.

*“I loved the fact that it – this is the first thing I’ve read where it’s captured the children’s views, like the voice of the child.” [PHP 3]*

*“I just think it gave a really good alternative view and the child’s voice there and for parents to kind of go ‘actually yeah maybe I’m thinking about it from my perspective, and not the child’s’.” [PHP 12]*

Overall, this meant the guidance provided reassurance to parents that they could talk about weight without harming their children.

*“This is giving you permission to say ‘it’s okay to say that word, it’s okay to say weight’.” [Parent 1]*

*“It sort of destigmatises it really doesn’t it, because it’s giving you permission.” [PHP 13]*

#### Reducing blame

Both parents and PHPs spoke of parents feeling “*blamed*” and “*guilty*” about their child’s weight status, and all felt the guidance dealt with this positively. Advice to avoid blaming oneself or children was perceived as effective because the recognition of environmental and external factors that can influence weight captured relevant challenges for parents. The message helped them feel that it “*isn’t somebody’s fault”*.

*“Yeah, it can be very easy I think, that blame game, to blame one particular family member, the person who does all the cooking or buys all the food, yeah, it’s important to have that in there because I’m sure a lot of parents do, I certainly feel guilty about my child.” [Parent 2]*

*“Quite often parents will say like ‘well I haven’t got the time’ or ‘it’s hard money-wise’ so I think putting that in there, about how hard it is sometimes to stay healthy was good, it took away that blame straight away.” [PHP 10]*

Many parents reflected on their own weight and experiences of trying to lose weight, and PHPs commonly reported that parents discuss their own weight when talking to professionals about their children. Content directed at parents who are struggling with their own weight was therefore considered very relevant, and the framing of children’s challenges as likely to be different from a parent’s was perceived as useful. Both PHPs and parents described this section as increasing their confidence in the possibility of a good outcome for children, and PHPs believed this would help to relieve parents’ feelings of guilt.

*“It can be difficult to feel confident […] if you’ve got your own weight issues or health issues, it can be hard to take the right approach. But I think the entire document gives you enough pointers to take that good approach.” [Parent 2]*

*“We do get a lot of parents where they’re already struggling with their own weight and they don’t necessarily feel that they’ve got the skills. So it’s giving them that confidence and the information there to say that they can do it together.” [PHP 12]*

### Theme 2: empowering parents: “it’s okay to say weight”

#### Boosting confidence

Both groups suggested that the guidance could improve parents’ confidence and make them feel more “*empowered*” about having weight conversations. Parents picked up on the message that there is no right or wrong way to talk about weight and the advice to use everyday opportunities to raise the topic. These aspects appeared to make having conversations with their children feel less daunting. The case studies seemed particularly helpful in this regard.

*“I think what is good is that it’s okay to do this any way, whatever way you feel as a parent is the right way to do it, is the right way to do it. We’ve given you three different stories of three different approaches and it’s not to say that any of them are right or wrong.” [Parent 1].*

The guidance also suggested framing discussions around weight and weight-related behaviours as being focussed on growth, health and energy. Parents reported that this framing gave them greater confidence in tackling weight conversations.

*“I did like the wording about, like, ‘let’s be healthy, let’s be active’ rather than the conversation always being about weight and being overweight and being heavy or anything like that. I did like that sort of positive angle on the language.” [Parent 2]*

Although not everyone perceived the advice as ‘new’, some parents who were already conscious of how they discuss weight and health were reassured to see their approaches affirmed by the guidance.

*“I find it quite reassuring to read something that just reinforces what you think you’re doing.” [Parent 5]*

PHPs felt the guidance would remind parents how influential they are in their children’s lives, and importantly thought it did this without suggesting blame or judgement.

*“I felt it was a leaflet that was good at boosting confidence. It was saying to me ‘you can do it, you can do this’. It had, for me, a good balance of acknowledging the challenges but also making it straightforward.” [PHP 4]*

Some parents reported previously wanting to talk to their child about their weight, but not having done so through lack of confidence in how to do it well. After reading the advice, some of these parents felt confident enough to try out the suggestions with their children before their interview.

*“It just gave me the confidence to speak freely and yeah, open up those channels of conversation.” [Parent 1]*

*“It definitely prompted me to have conversations with her that I’ve needed to have, so it’s been really positive.” [Parent 11]*

#### General versus specific advice

Despite the guidance being perceived as reassuring for most parents, some found the advice too general and felt it lacked applications or recommendations about talking to a child specifically about their weight and what it means to be overweight.

*“I probably wanted either a section or the guide to be […] ‘how do I talk to my daughter about being overweight’? Not just, talking to her about weight.” [Parent 10]*

Some parents would have welcomed reassurance and advice on what to do if their child refuses to engage in conversations about weight, and suggested including this as a specific scenario.

*“It might also be helpful to have an example of a child that doesn’t want to talk about it and how to support those because there will be children out there that don’t want to talk about it, but parents need help on how to approach that as well.” [Parent 6]*

While others recognised why the guidance does not provide prescriptive advice, this was found to be disappointing.

*“I was a bit disappointed. Because I think I was hoping for a miraculous answer that I haven’t spotted. […] Or – I realise it’s not like this – but almost a set of rules to say ‘do this, do this and do this’ and everything will be fine, but of course it’s not like that, but that’s just my feeling.” [Parent 8]*

### Theme 3: believability and achievability

#### Making realistic changes

Parents felt the content and language in the guidance was realistic and “*resonated”* with their experiences. The section ‘responding to difficult questions’ was perceived as helpful because many parents could relate to the scenarios and felt the suggested responses sounded natural and normal. This section included specific examples of common questions or scenarios that parents may encounter, along with several alternative responses to consider.

*“I felt like everything in there I could take something from. There was a couple of sections that resonated to me, I was like ‘oh that’s exactly what I need’. The difficult questions section, that one was a real big one for me that was really helpful.” [Parent 1]*

Both parents and PHPs reflected on how parents can struggle to “*know what to say”* and thought the specific ideas and examples of conversation starters would help overcome this. PHPs also perceived this section as useful as it covered issues that commonly arise in their practice and they felt it offered parents quick, practical reminders.

*“Those little tips of how parents could respond I thought was actually really quite useful because it’s something that perhaps parents could go back to and look at when that scenario arises, rather than refreshing themselves on the whole document.” [PHP 9]*

Both parents and PHPs commented that advice around promoting health, openness and kindness was useful because this could be applied to other parenting topics.

*“I think these are sort of things to think when you’re talking about anything I think with kids. […] It’s relatable to different conversations that you might have about different things.” [Parent 2]*

*“I thought it was a really useful tool with a really good parenting kind of ethos running through, it wasn’t just about weight, there were some really good parenting suggestions in there as well.” [PHP 10]*

Further, PHPs felt that the framing about health aligned with their current advice to parents. They endorsed the encouragement of taking a ‘whole family approach’ to healthy lifestyles by involving children in making healthy choices.

*“A lot of the way through this the information reinforces the messages that we do try and give as health professionals, so I kind of like that it reinforces that really.” [PHP 12]*

While most responses were positive, PHPs also recognised that the guidance may not be relevant to poorer and more disadvantaged families, and that this could be improved by including more relevant example scenarios or making existing suggestions more inclusive.

*“We do have families where there won’t be a choice of what they can have. […] The areas that we work in are quite deprived areas, so they’d never probably choose fruit and veg, but they might want to.” [PHP 6]*

Similarly, several parents and PHPs suggested developing separate sections for different age groups to improve the usability of the guidance. For example, parents of older children may benefit from further advice on dealing with the impact of social media on weight, whereas parents of younger children may find suggestions around making fun, healthy changes more relevant.

#### Modelling

The case studies stood out as particularly believable and effective elements because parents could identify with the characters and relate the situations to their own family. The narrative style was reported to be engaging and made the advice seem more “*honest”* and “*personal”* than straightforward advice. Identification with the characters reassured parents they are “*not alone”* in their struggles and demonstrated positive role models of parents making changes with their children.

*“Yeah at the moment that’s where I’m at, I’ve got teenage children and I can’t control what they eat all the time but it’s empowering them to make good choices. And I think this case study shows how they can do it all together as a family which is really good.” [Parent 9]*.

*“If you don’t want to talk about it, you don’t have to, but you can really change as a family. I think a lot of parents will relate to that, it’s probably gonna bring the family together, they are gonna be doing things together, going for walks and things.” [Parent 6]*.

PHPs also considered the case studies to be credible and relatable, recognising that sharing other people’s stories is an effective way to convey information and prompt reflection.

*“Each story addresses a barrier to the reason it’s come about, the reason for the story, and then how that barrier was overcome in a way that I think will be quite reflective of real life that I think parents will relate to.” [PHP 4]*.

## Discussion

This study explored parents’ and PHPs’ perceptions of new parental guidance on talking to children about weight, resulting in three key themes. Theme 1 shows how the guidance helped overcome several barriers to parent-child communication: it helped parents reflect on why they avoid talking about weight with their child, and reduced perceptions of blame; the information provided reduced parents’ uncertainty and anxiety; and finally, the guidance reassured parents that both they and their child had the potential to benefit from talking more openly about weight. Theme 2 shows how parents and PHPs felt the guidance boosted parents’ confidence to attempt conversations they had been wanting to start, or affirmed their current approaches, which they described as empowering. Theme 3 covers how and why different sections of the guidance were found to provide realistic and practical information to parents. Across all themes, the response to the guidance was predominantly positive. However, some limitations were also raised, including the inclusivity of the guidance for families living on lower incomes or in more deprived areas, the need for different advice for different ages, and the perceived lack of advice on talking to children about the child’s *own* weight, particularly for children with overweight, rather than weight in general.

### Mechanisms of action

The guidance was designed by mapping elements of its content (techniques, style and tone) to specific functions these were intended to fulfil, including: reducing negative affect and emotional responses; improving knowledge and skills; and improving parents’ confidence to initiate a positive conversation with their child about weight [25]. Insight from the extracted themes allows us to consider whether the parents we interviewed considered that these functions were met.

#### Reducing negative affect

Our interviews echoed previous research suggesting that many parents feel they are to blame for their child’s weight, which contributes to feelings of shame and stigma [38]. Theme 1 reports how parents and PHPs responded to the techniques we had included to address this. For example, acknowledging environmental factors in the development of obesity and giving a clear recommendation that families avoid blaming parents or children for their weight status, as suggested by past research [28–30]. Respondents felt these strategies could reduce stigma and there were examples of parents relating this to their child (e.g., acknowledging that talking about weight at home might prevent it feeling like something too shameful to mention). Parents also talked about feeling less “*frightened*” to have conversations about weight after reading the guidance.

During the design phase, stakeholders talked about the challenges parents feel in addressing a child’s weight when the parents are overweight themselves, and how this can undermine the belief that it is worth making changes. The guidance acknowledged this by encouraging parents to disentangle their own weight status and history of managing their weight from their child’s. PHPs in particular felt this would be useful and likely to be effective in reducing feelings of guilt.

#### Improving knowledge, skills and confidence

The messaging that there is no right or wrong way to talk to children about weight and health (raised in Theme 2) was felt by respondents to potentially increase parents’ confidence. Providing believable case studies and usable tools, in the form of credible wording, framing and suggestions on when and how to raise the topic, were also considered to boost confidence; Theme 3 presented how the narrative case studies could do this by prompting parents to engage in cognitive elaboration and reflection (i.e., thinking about how they would react in the same situation and how this might apply to them now). Previous research supports the role of cognitive elaboration in prompting behavioural changes with narratives [39–41]. Parents appeared to identify with the characters in the case studies, reported noticing novel ideas and new perspectives from reading the accounts, and suggested that reading about positive results for the characters in the case studies boosted their own self-efficacy.

### Potential refinements and future directions

PHPs suggested modifying some examples to be more realistic for parents with limited resources. For many families, healthy food is unaffordable and opportunities for physical activity are limited. Given that children living in the most deprived areas of England are more likely to have obesity than those from the least deprived areas [2], and that engaging parents from deprived areas in public health interventions can be challenging [12, 15], ensuring that the guidance is relatable and useful to more disadvantaged families is crucial. We did not collect data on parents’ socioeconomic status or ethnic group (see Limitations), but further work should seek to engage parents from a range of backgrounds to determine the usefulness of the guidance across the population. Both parents and PHPs commented on how the length of the guidance had initially been off-putting. However, they felt that all content was necessary and found the length was not a problem once they had started reading. Future development should explore whether alternative formats which break down the guidance into smaller sections might help to reduce this (e.g., through a website or a set of learning modules).

Both PHPs and parents also commented that receiving the guidance at the point of NCMP feedback may be too late, and that early familiarity with the content may be more helpful. The relevance and perceived utility of providing the guidance at other times, for example at key points of contact with primary care or through other freely accessible routes, may be useful to explore.

The guidance has now been produced for general use, available through the British Dietetic Association [42] and lead author’s institutional websites [43]. Online versions are being incorporated into national public health online provision for parents, alongside guidance for promoting a healthy weight, for example by the Scottish Government [44].

At this early stage of development, the guidance has only been made in English language format and the acceptability of this format only was explored in the current study. Future work is now needed to adapt the guidance to best suit the needs of parents and families from different cultural backgrounds living in England, bearing in mind that effective cultural adaptation requires more than simple language translation [45].

### Strengths and limitations

The strengths of this study included the recruitment of participants from multiple local authority areas in England, reflecting variations in demographics and practice with regards to the provision of NCMP feedback and availability of weight management programmes [17]. Including PHPs’ insights allowed us to explore how the guidance could fit with their work, both in terms of whether it meets their needs regarding common concerns from parents, and whether it aligns to their accepted approach and practices.

Limitations of the study included the lack of men interviewed across both groups; while this is common among research with parents and reflective of the PHP workforce, it means men’s perspectives are not well represented here. Also, whilst PHPs provided insight on the experiences of families in their areas, we did not collect demographic information from parent participants. As such, it is not possible to draw conclusions regarding the suitability and acceptability of the guidance for parents from different backgrounds. Similar to other relevant research, the self-selecting nature of the sample meant that parents and PHPs in our study may have been those who were particularly interested and engaged with child weight and health [46]. Perceptions of the guidance may vary amongst parents and professionals with less interest in these areas, and among parents who are less accepting of NCMP feedback.

## Conclusions

Our findings confirm that guidance for parents on talking to children about weight is important and needed. Overall, parents and PHPs perceived the new guidance as acceptable, engaging, and useful. Feedback from parents demonstrated the anxiety and fear they can feel in relation to talking to children about weight, and the provision of guidance in this format appeared sufficient to alleviate that for some parents. The narrative case studies and inclusion of the child’s perspective were identified as particularly novel and effective elements, supporting their use in health interventions for parents. Potential refinements to the guidance were identified, including modifications to specific suggestions and creating separate versions for different age groups. Future research is warranted to explore the impact of the guidance on parents’ and children’s experience of the NCMP, in terms of reducing negative emotions, promoting positive, constructive conversations, and ultimately supporting changes to reduce excess weight.

## Electronic supplementary material

Below is the link to the electronic supplementary material.

Supplementary Material 1

Supplementary Material 2

Supplementary Material 3

Supplementary Material 4

## Data Availability

The datasets used and analysed during the current study are available from the corresponding author on reasonable request.

## List of abbreviations

LAs: Local Authorities
NCMP: National Child Measurement Programme
PHP: Public Health Practitioner
