# Supplementary material for: The acceptability of evidence-informed guidance for parents in talking to their children about weight
Source: BMC Public Health. 2023 Jul 14;23:1357. doi: 10.1186/s12889-023-16267-6 (PMC10349501; doi:10.1186/s12889-023-16267-6)
Supplement: Supplementary file 1 — Supplementary Material 1 [file 12889_2023_16267_MOESM1_ESM.docx]

**Additional file 1 – Parent Interview Schedule**

Thank you for agreeing to take part in this project. We are interested in your views on the new guidance resource we sent you, so in this call, I’ll ask you some questions about this. There are no right or wrong answers, we’re just interested in your opinions. I’ll record what is said in this call so that it can be typed up later, but any information that could identify you, such as names of people or places, will be removed. If at any time during the call you would like to stop, just let me know, and you do not have to answer any questions that you do not feel comfortable with.

Could you start by telling me a little bit about your family?

- How old are your children?

What kind of experience, if any, have you had in talking to your children about weight?

- Have you ever used any guidance like this before?
- If you haven’t talked about weight, why do you think that might be?

Thinking now about the guidance we sent, what were your initial thoughts on it?

- Any first impressions?
- What stood out to you?

Could you tell me about any aspects of the guidance that you particularly **liked or disliked**?

- What were your thoughts on the length of the guidance?
- What were your thoughts on the use of pictures?

What did you think about the stories in the guidance?

- How did they make you feel when reading them?
  - Was there anything in particular in the story made you feel that way?
- How relevant or relatable did you find them?

Did the tricky scenarios and responses at the end of the guidance cover things that happen in your family?

- Does the wording of the responses sound like something you would say? How would you put it if differently?

How do you feel about having these conversations with your child after reading the guidance?

- Has it affected your confidence to talk with your children about weight?
- Why do you think that is?

How well do you think the topic of overweight is covered?

- Are there any parts that are particularly good/bad at covering overweight?

What formats would you prefer to see the guidance in? (e.g. leaflets, on a website or app)

- At what times or places would it be most useful for you to see this? (e.g. school, GP surgery, pharmacy)

Have you recently had a feedback letter from when your child was weighed in school?

- If yes, would this have been useful alongside that, and if so, would it be more useful before or after that feedback?

Overall, would you find this guidance useful?

- How likely would you be to recommend the guidance to another parent? Why/why not?

That’s all of my questions but do you have any further comments or suggestions about the guidance?
