# Supplementary material for: The acceptability of evidence-informed guidance for parents in talking to their children about weight
Source: BMC Public Health. 2023 Jul 14;23:1357. doi: 10.1186/s12889-023-16267-6 (PMC10349501; doi:10.1186/s12889-023-16267-6)
Supplement: Supplementary file 2 — Supplementary Material 2 [file 12889_2023_16267_MOESM2_ESM.docx]

**Additional file 2 – Public Health Practitioner Interview Schedule**

Thank you for agreeing to take part in this project. We are interested in your views on the new guidance resource we sent you, so in this call, I’ll ask you some questions about this. There are no right or wrong answers, we’re just interested in your opinions. I’ll record what is said in this call so that it can be typed up later, but any information that could identify you, such as names of people or places, will be removed. If at any time during the call you would like to stop, just let me know, and you do not have to answer any questions that you do not feel comfortable with.

To start with, can you tell me what contact you have with families of children with obesity/overweight?

- Can you describe how things work in your area (e.g., are there child weight management services to refer into)?
- How long have you been working with families in relation to childhood weight and obesity?

What advice do you currently give to parents in how they talk to their child about weight? (and where do you get this information from?)

- Are there any resources that you currently direct parents to?

Thinking now about the guidance we sent, what were your initial thoughts on it?

- Any first impressions?
- What stood out to you?

Could you tell me about any aspects of the guidance that you particularly **liked or disliked**?

- What were your thoughts on the length of the guidance?
- What were your thoughts on the use of pictures?

Do the issues covered in the guidance align with what you talk about with parents yourself?

- How relevant/relatable do you think they will be to the parents that you work with?

Did the tricky scenarios and responses at the end of the guidance cover things that happen in the families you speak to?

- Does the wording of the responses sound like something the families you speak to would say? How would they put it if differently?

Would having this guidance make you more confident/positive about talking to parents about their child’s weight?

- Why do you think that is?

How well do you think the guidance deals with issues of weight stigma?

- Are there particular parts that should be changed?

What format do you think would work best for parents to receive the guidance? (e.g. paper leaflets, online website or app)

- When and where do you think this would be the most useful to parents – particularly thinking about before or after the NCMP, but other times or places too?

Overall, do you think this guidance would be useful? Why/why not?

- Would you recommend this to other people you work with, and if so who?
- How can you see yourself using this in your practice?

That’s all of my questions but do you have any further comments or suggestions about the guidance?
