# Supplementary material for: The acceptability of evidence-informed guidance for parents in talking to their children about weight
Source: BMC Public Health. 2023 Jul 14;23:1357. doi: 10.1186/s12889-023-16267-6 (PMC10349501; doi:10.1186/s12889-023-16267-6)
Supplement: Supplementary file 3 — Supplementary Material 3 [file 12889_2023_16267_MOESM3_ESM.pdf]

Talking to your child about weight

A guide for parents and caregivers of children aged 4-11 years

The aim of this guide is to help parents and caregivers talk with their children about weight in a positive way. It gives tips and advice on what to say and do to help children be healthy and feel good about their bodies.

This guidance is for parents and caregivers of children of all shapes and sizes.

CONTENTS

SECTION 1: Should I talk to my child about their weight?

SECTION 2: The whole family counts

SECTION 3: Top tips for talking to your child about weight

SECTION 4: How to help your child feel good about their body

SECTION 5: What if I am struggling with my own weight?

SECTION 6: What could I say when…?

SECTION 7: Extra advice and information

Prepared by:
Fiona Gillison,
Elisabeth Grey,
Angel Chater,
Lou Atkinson
and Alison Gahagan

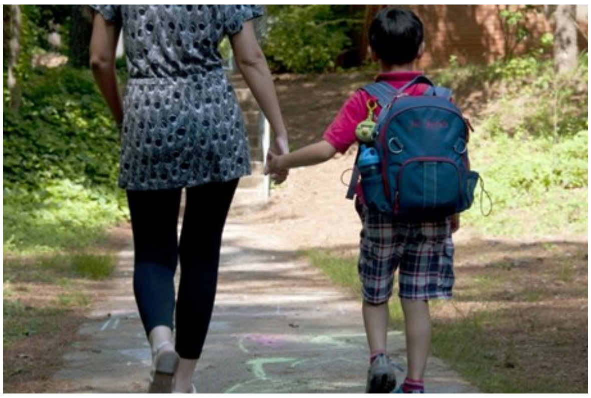

In this guidance, to keep the text short, we use the word 'parents' to mean both **parents** and other primary **care-givers**, like legal **guardians** or **grand-parents** providing informal care.

Public Health England

UNIVERSITY OF BATH

Section 1: Should I talk to my child about their weight?

- There are times when you may decide it is useful to talk with your child about their weight.

- Talking openly about weight rather than avoiding the topic can help to **build trust** and stops it feeling like something to be ashamed about.

"If it's a problem it needs to be talked about, but if you're healthy that's also great news, so I think that's an important thing to discuss." ***(Isla, aged 11)***

- Children want to know if they are overweight or not and want their parents to **help them to be healthy**.

"Your parents can give you information on how to improve your weight and how to build your weight up properly." ***(Yusuf, aged 9)***

- Children will hear about weight from many different places – in school, from friends, on TV, online. You can help them to see that **what they hear about weight is not always right**. Remember that children trust their parents.

- Talking to you about their weight could be your child's only chance to ask questions openly and learn about their weight and health.

"If parents don't talk about weight, children might think that their parents won't like them if they're not the right weight." ***(Leo, aged 9)***

- Talking with your child about their weight in a **kind and supportive** way can help them feel good about their body. The advice in this guidance aims to help you do this.

- The most important thing is that your child feels able to talk to you.

Section 1: Should I talk to my child about their weight?

Things to remember

- You have a big influence on how your child thinks and feels about their weight.
- Children know that their weight is a sign of whether they are ‘growing properly’ and expect parents to talk to them about this. Talking about weight may be a bigger deal to you than it is to them.
- Letting children know that weight is something they can talk about - and is just one part of how we know if they are healthy - can help to reduce shame around weight.

Avoid blame

No one gets it right all the time so try not to blame your child or yourself for being a certain weight

– focus instead on the healthy things you and your family can do.

The way we live now makes it hard for all of us to stay healthy – it isn’t easy to eat well all the time and make physical activity part of daily life.

Talking to children about what things might push us to make less healthy choices can help them make healthier choices for themselves

—you could talk about things like food adverts, shops putting sweets near the checkouts, and taking the car rather than walking.

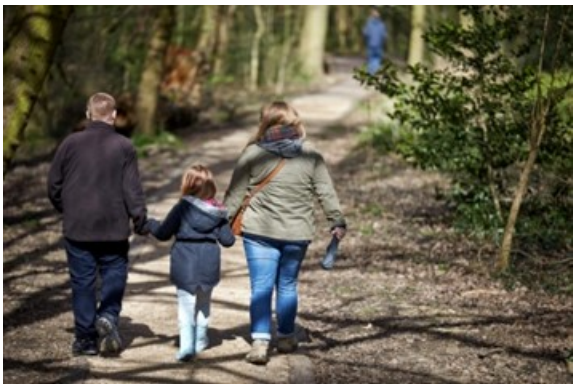

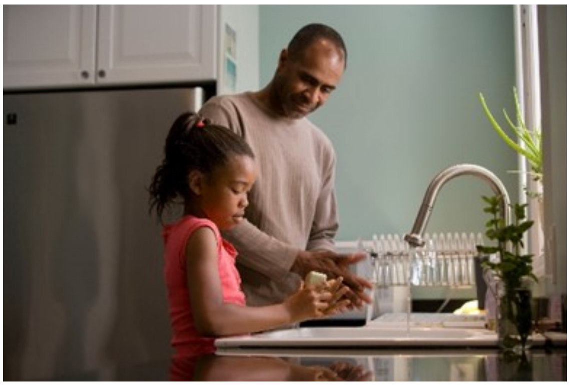

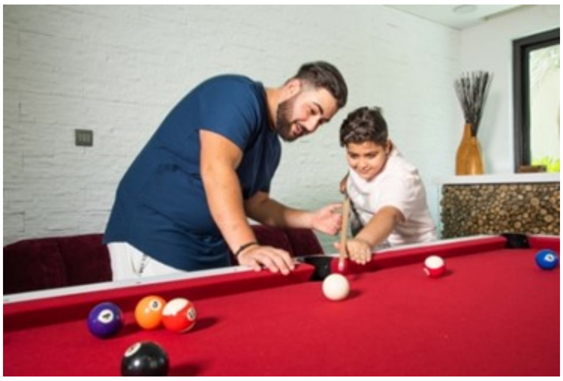

Mark's story

Mark was 11 and **already knew he was one of the heavier children at school** before the measurement day came along – not least because the other children sometimes teased him about it. **He didn't really know whether or not it mattered for his health, or whether he could do anything about it** as his parents never talked about weight at home. But it made him feel bad, and he thought it was probably also the reason why he couldn't keep up with his friends during PE. So although it was a bit embarrassing to be weighed at school, it was also quite a relief as he thought this would mean someone would help him with it.

Mark knew he was one of the heavier children at school

Mark was really disappointed when his mum didn't mention his weight after the measurements in school. **It felt to Mark like being overweight was so shameful that no one was willing to talk about it.**

Things changed for the better after his mum started talking to him about healthy eating as part of his school homework one day. She asked him whether he thought their family was healthy, and what he knew about healthy eating. After that Mark found it easy to tell her that he was worried he was too big, and that sometimes the other kids teased him about his weight. **His mum was really helpful and sat down with him to talk about what different things they could do together to be healthy.**

She also talked to his school teacher who started doing some activities in class to help all the children treat each other with a bit more kindness. His mum made sure his teacher didn't mention Mark of course, but he found the teasing soon stopped after that.

Talking with his mum about his weight made things better

One thing Mark and his Mum decided to do was join a local course for children and their families who wanted to be healthier. He was a bit nervous about this, but found he really enjoyed it. There were lots of games and activities to learn about changes they could make that would help their health. Even though he realised that he wouldn't reach a healthy weight quickly, knowing that he was becoming healthier made him feel a lot more confident .

Mark's mum made some healthy changes at home too for all the family, like trying some new recipes. They didn't always like them, but his mum said they hadn't always liked what she cooked for them anyway so it was no different!

For more information about finding support, **see Section 7**

Section 2: The whole family counts

What you do and say counts

Your child learns from you all the time and will copy what you do. What you say, what you do, and how you talk about yourself and others are very important in teaching your child about weight and size.

Children learn to judge people by their weight and size if they see or hear others doing it - *you can't control everything they see or hear, but you can try to:*

- Avoid criticising your own weight or appearance and that of other people – this can make children think this is how you will judge them too
- Greet people by saying how nice it is to see them rather than with comments about their appearance
- Talk about making changes to help your child grow, be healthy and do the things they want to do (play, learn etc.) rather than to control weight

Talk positively about food and physical activity

- Help your child to learn that eating a range of foods and being active are normal and enjoyable, not things to be done only to control our weight
- Try and make sure your child sees you eating a range of foods and being active yourself—most of the time!

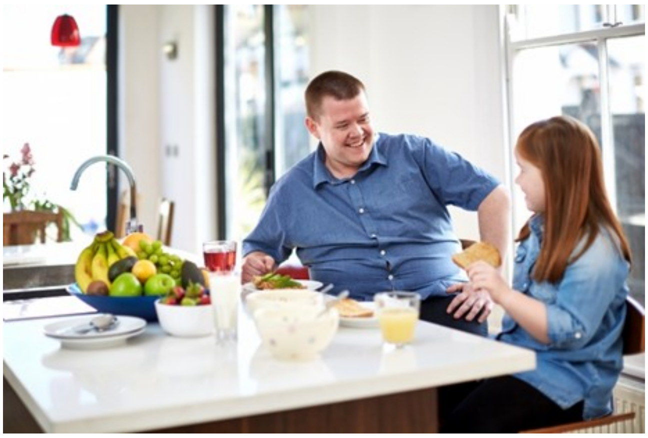

What could I say…?

"Great, you've eaten all your vegetables, those will help you be healthy and grow well"

"I feel better after that walk, don't you?"

"What do you think we should choose for tea, what can we eat to keep us all healthy?"

"The park will be wet in the rain today – shall we go and see how many puddles we can find?"

Section 2: The whole family counts

Get other adults to help too

Children understand better if they get the same message from everyone.

Mothers, fathers, grandparents and carers can ***all*** have a big impact. You can support each other.

Try talking to other adults who care for your child about how you will talk about weight – could you agree on some rules (like not criticising the way people look or their size)?

Parents don't always agree with each other – but you can still make sure the messages *you* give to your child are always the same.

You can ask older children what they think about their parents having different ideas - after all, they will hear different views outside your home too.

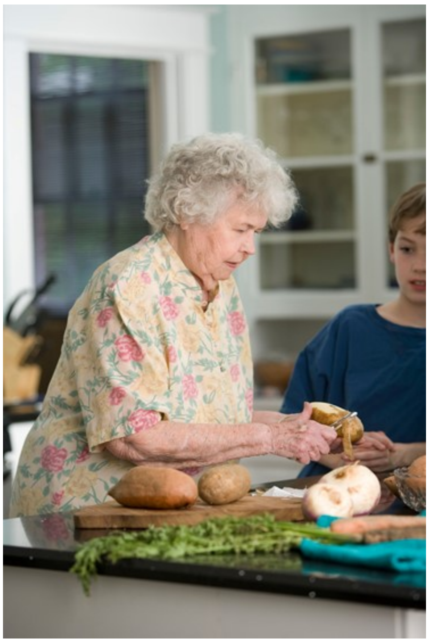

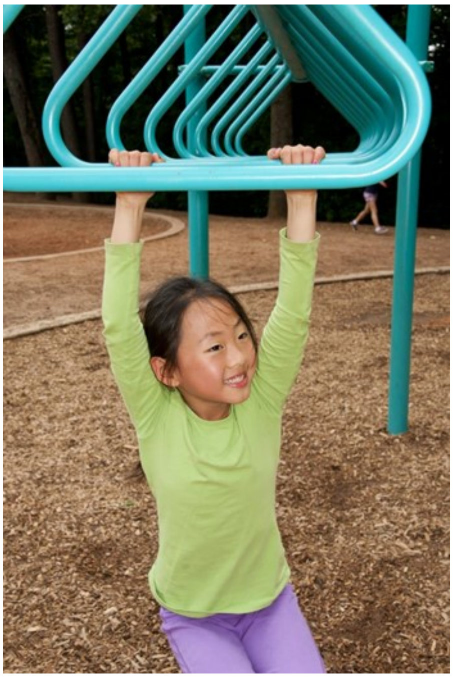

Choosing changes together

Giving children choice where you can, and involving them in making new habits may help to keep them working with you, rather than pushing back. You could try:

- asking your children to pick between 2 healthy options to make for dinner
- allowing your children to choose fun physical activities for the family to play
- letting them have their say when agreeing rules for screen time.

Making changes as a whole family can feel easier and be fun!

Ruby's story

Ruby and her classmates in Reception were excited when the nurse visited their school to measure their height and weight. The nurse said the measurements would be sent in a letter to her parents.

After school that day, Ruby told her dad about being measured and asked whether he would tell her what the letter said when it came. Ruby's dad agreed but he felt a bit nervous – **they never really talked about weight because he didn't want Ruby to start worrying about her appearance.** He had never talked about weight with his own parents or with his friends, so he just wasn't sure what to say. He tried to be honest when Ruby had questions about other things and that seemed to work alright – she was a happy and confident girl - so he guessed he should take the same approach with weight.

Ruby's dad wasn't sure what to say—he had never talked about weight with his parents

If children, like Ruby, are curious about their weight, talking to them about it can help check if they have concerns.

The letter came a few weeks later and Ruby's dad told her what it said, as he had promised. "*It says that I'm a healthy weight, so does that mean it's good*?". "*Well, yeah, that means you're growing well, and I know you eat healthy food – mostly! – and you're very active, which will make you strong*" replied her dad. "***But most importantly you're happy. Now, did you want to ask me anything***?". Ruby didn't, she was really just curious about her results. Her Dad was relieved to know **Ruby wasn't worried about her weight or health,** and felt better having checked.

Many parents are unsure how to talk about weight with their children – we may have little experience of talking about weight or we may have concerns about our own weight that we don't want to pass on. But children are curious and may have questions that you can help answer. ***Letting your child know that they can talk to you about weight can stop them worrying.***

Not all areas will send out results letters to parents from the school measurement programme. Your school nurse will be able to tell you what will happen at your child's school.

Section 3: Top tips for talking to your child about weight

How could I start the conversation?

Small conversations can work better than one 'big' talk

- Talk about growth and health where it comes up **in everyday conversations** and focus on these rather than weight itself
- Focus on the things your child can do to look after their health (**keep active, eat their greens etc.**)
- Don't feel you have to talk about everything in one go

Pick times when it's more natural to talk about food, activity or weight, for example:

- when cooking or food shopping
- reading a cereal box over breakfast
- when it comes up on TV
- when shopping for clothes
- when a child talks about their own or someone else's size
- when talking about what your child has done at school.

What could I say…?

"What have you learnt at school about how to be healthy ?"

"I'm really looking forward to our walk together"

"Where do you find out about what is healthy – is it at school, from the telly, or other places?"

"What could our family do to be more healthy?"

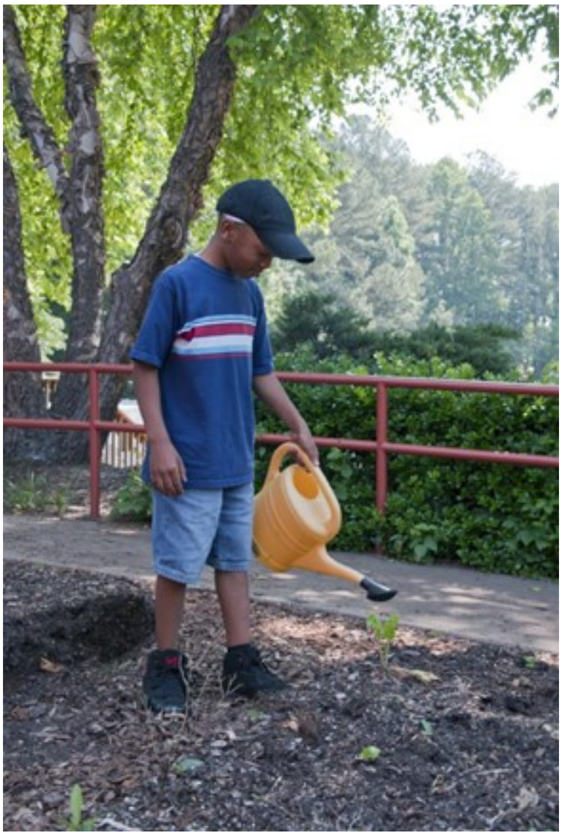

Section 3: Top tips for talking to your child about weight

What words should I use?

Some people prefer not to use the word *'weight'* but to talk about *growth* and *health,* or *exercise* and *healthy eating*. If this is what you and your child are comfortable with, this is fine.

But sometimes it can be helpful to talk about '*weight*', for example, if:

- your child asks about their own or someone else's weight or size
- you hear your child use hurtful words about someone's size
- other people, including health professionals, talk about weight with your child
- you get a letter from the National Child Measurement Programme about your child's weight
- your child mentions talking about weight or weight teasing at school – take this as a chance to ask them what they think about weight and how they talk about it with their friends.

'Weight' shouldn't be a banned word as this can create shame and worry, so talk openly about weight if your child wants to.

Be confident

Children want to understand about health, growth and weight, and they know you want what is best for them.

Children look to their parents first for guidance

Parents are really important in helping children make sense of things.

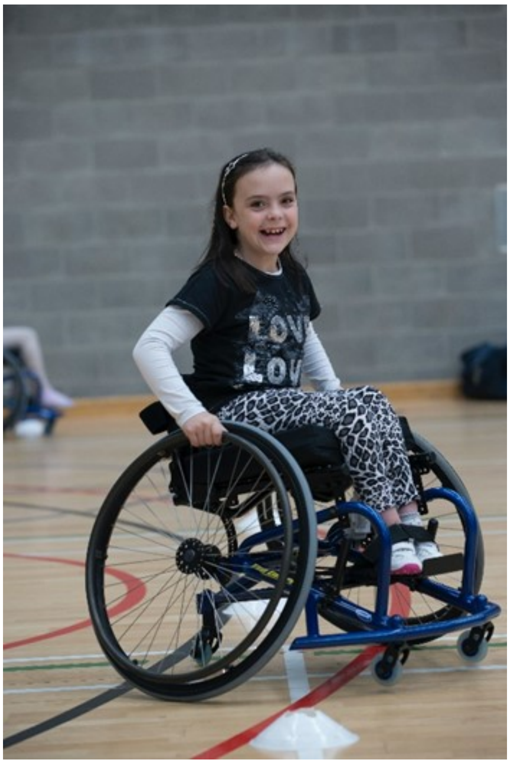

Section 3: Top tips for talking to your child about weight

Talking about 'weight' in particular

- Find a time when other siblings are not around if you think your child may be worried or embarrassed about their size.
- Use open questions (avoiding yes/no answers) to encourage your child to talk about what they have heard or may be worried about. For example, "what do you think about….?" instead of "Are you worried about ….?"
- Talk about weight being important for health, energy and what the body can do.
- Explain that we cannot know what a person is like based on how they look – just because someone is thinner does not mean they are kinder or more hard working than someone who is larger.
- Make time for your child to ask questions.
- Let your child know they can talk to you about weight or size if they want to, but don't push them to talk about it if they choose not to.

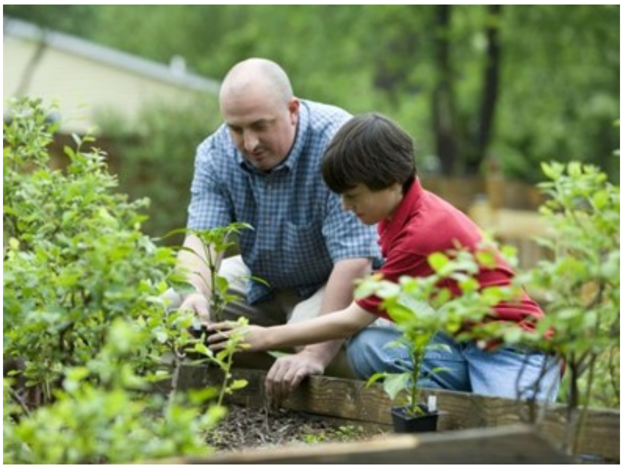

What could I say…?

"Is there anything you want to know about weight?"

"Why do you think doctors and nurses think your weight is important?"

"How do you think it would feel if some-one called you 'fat'? What would be a kinder way to talk about someone with a larger body size if you needed to?"

"What do you think about your weight/ size? And what about the rest of us in this family?"

Remember…

The most important thing is that your child feels able to talk to you, and not whether you have the perfect answer straight away.

Sam's story

When Sam's daughter Aisha was measured in year 6, Sam and his partner got a letter saying Aisha was very overweight. Sam did not want to talk about this with Aisha but instead they decided to make some healthy changes together as a family.

"We've always been quite big in our family - no one would look at us and think for a minute we'd have really skinny kids, so I guess **we just didn't really notice when our middle child, Aisha, started to put on weight** in primary school. She'd always been one of the bigger kids in her class but not the biggest.

When I got this letter telling me she was very overweight it was a real shock and, to be honest, I was really upset.

I was really upset when I got the letter

I already thought I was doing all I could, making sure they all had some veg at dinner and didn't stay on the computer all night and all that. I wasn't going to start being so strict that I made their lives miserable.

Also, I suppose because I'**ve struggled with my own weight all my life**, I didn't really believe that anything I could do would make a difference - so it was better not to risk making her self-conscious about it.

So to start with I wasn't going to do anything different after getting letter.

It was my partner who snapped me out if it, saying **'*why don't we just all try getting healthy as a family*?'**

I was determined that we wouldn't do anything drastic, and I absolutely didn't want to single Aisha out and make her feel different from the other kids. But doing things together felt alright.

I didn't want to single out Aisha — we've made changes as a family

Not everything has worked, but some things have, like having different fruit for snacks after school instead of biscuits, and I get the kids to help me find healthy recipes online to try for dinner. We've also started going for walks together at the weekend – the kids aren't always keen to leave their screens and if it's cold, neither am I! But **we all usually enjoy it once we get outside, especially if I distract them with a game.**

It's been hard, though, to find the right line between making the family healthier, and **not making it a big 'thing'**. I never said anything to Aisha about the letter, I know what it felt like to be told I was 'fat' as a child.

***Aisha*'s 13 now, and I can't be in charge of what she eats or does every minute of the day**, so we're just trying to do our best with talking about healthy eating and being active at home so at least she understands what the choices are. Actually, in her new school she buys her own lunch, and normally chooses something quite healthy - so maybe it is working!"

SECTION 4: How to help your child feel good about their body

- Teach children that everyone deserves respect, whatever their body size, shape or ability - this will help them not to worry about their own body too

- If you talk about your child's weight, let them know it's because you want them to grow well and be healthy, not to look a certain way

- Talk about the amazing things our bodies can do, regardless of size

- Avoid saying that your child or other people should do or wear certain things because of their weight

- Praise your child for a variety of things so they know you love them for who they are, not what they look like or only when they do well at something

- Talk to your child about what they see online, social media and on TV – explain that lighting, make-up and photo-editing is used to make people look different from how they are in real life.

- Children (and adults!) are more likely to keep doing things if they enjoy them — try and help children notice the benefits of exercise, like fun, energy and feeling good, and find the kinds they like the most.

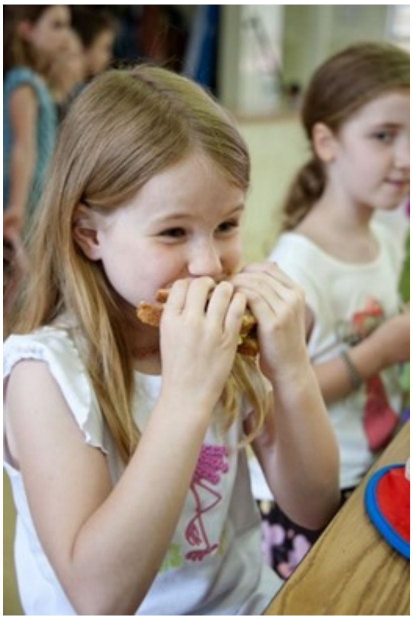

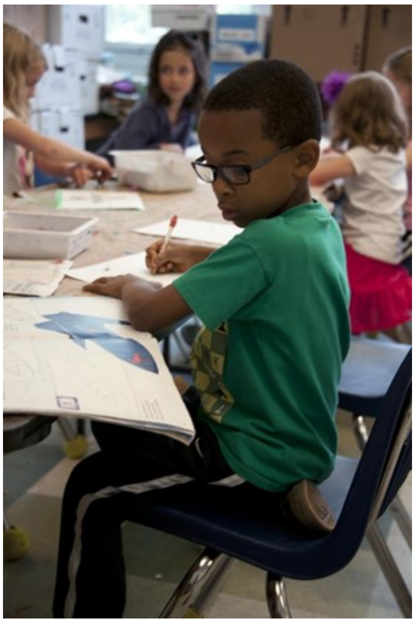

SECTION 4: How to help your child feel good about their body

What could I say…?

"It looks a bit like that picture has been photoshopped—what might have been changed?"

"I'm really looking forward to our walk at the weekend—it keeps us healthy and feels great to be outside!"

"All bodies are different and that's ok."

"You've had a really busy day today, isn't your body amazing to keep you going with so much energy?"

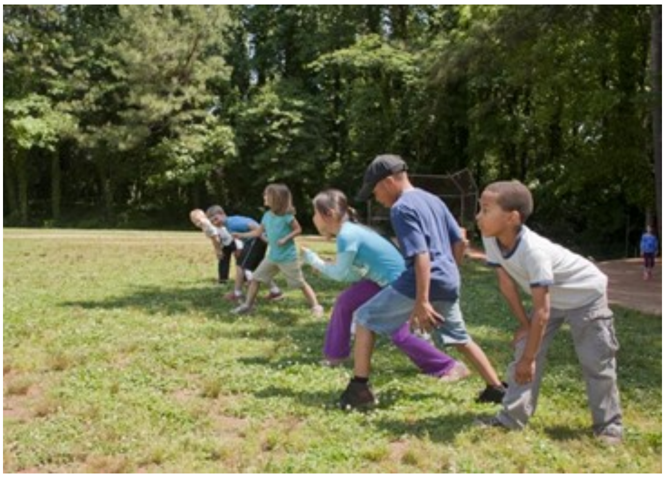

"They know I'm only teasing…"

Some words and phrases are said 'affectionately' or 'playfully' - but they can still be **hurtful even if your child seems to laugh it off**.

Encouraging children to enjoy healthy eating and being active is more likely to work than teasing or punishing them.

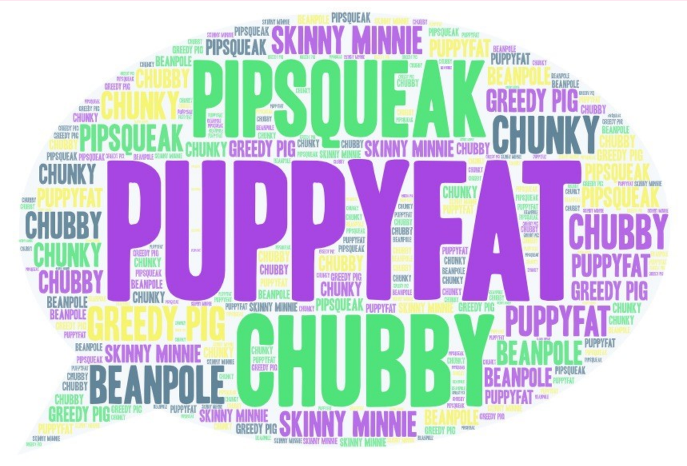

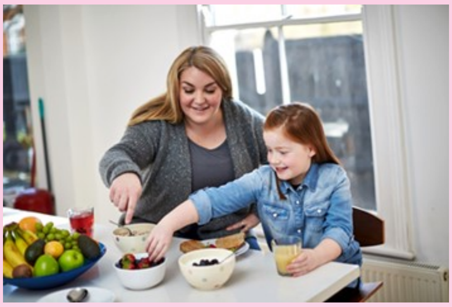

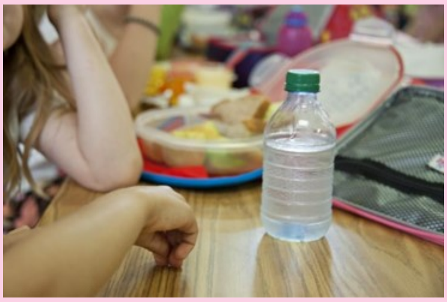

SECTION 5: What if I am struggling with my own weight?

If I'm not happy with my weight, how can I help my child with theirs?

Many adults are unhappy or struggle with their weight. But whatever your weight, you can still help your child reach a healthy weight.

Your child's experience will be different from your own—they may not feel the same way as you do, or as you did as a child.

If you are trying to lose or gain weight yourself:

- avoid talking about diets and dieting

- show that healthy eating is normal and important for everyone, rather than eating different foods from the rest of the family

- do physical activity that you enjoy - this will help you manage your weight and your children will see you enjoying activity, rather than treating it as a chore.

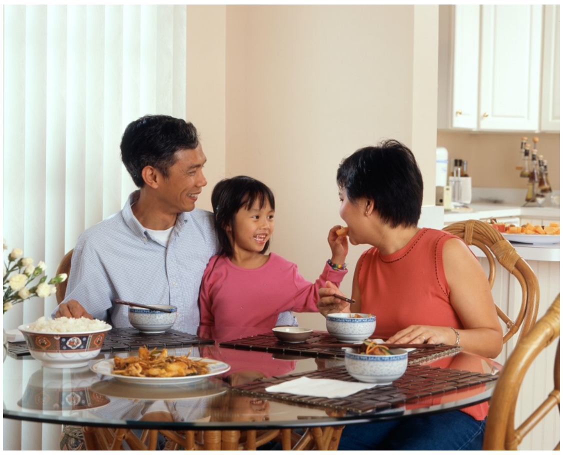

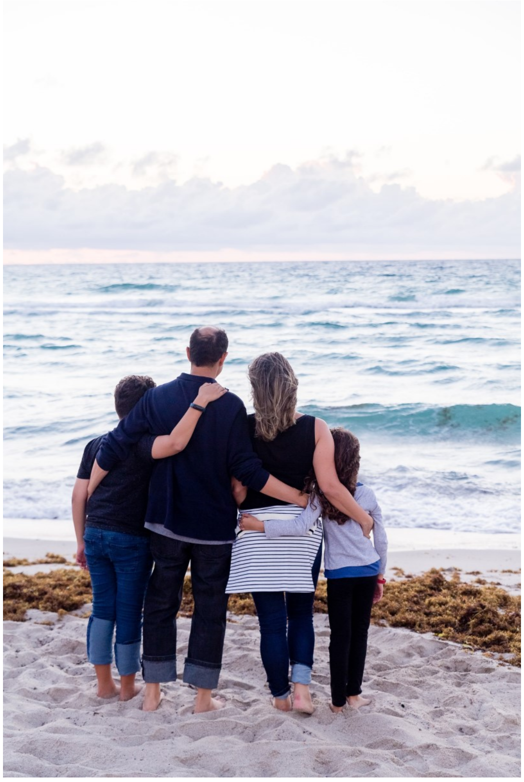

SECTION 6: What could I say when…?

Below are some common situations that parents have told us can be hard to deal with – along with suggestions of responses to try.

1. Your child asks why you are exercising (especially if it's new to you)

Try responses like:

"Why do you think I'm exercising?" *Listen to your child's answer and add positive reasons to what they suggest*. "Because it makes me feel good/relieves stress/helps me to keep up with you"

…*and if you are doing it to lose weight, and want to tell your child this, how about*: "I'm doing it to help me to be more healthy and feel fitter"

2. Your child comments that someone in the street or on TV is "so fat!"

Try responses like:

"I guess he/she is larger than some people, but everyone looks different"

"How do you think they would feel if they heard you saying that?"

"Whatever their size they may be just as clever/friendly as you are, and that's much more important than what they look like"

3. Your child says that someone called them fat in school

Try responses like:

"That sounds a bit unkind. How do you feel about it?"

"It's not good to judge people on what they look like – what really counts is what you say and do - being kind and friendly, like you are."

"Do you want to talk about what you could do if it happens again?" *Talk through ideas such as telling a teacher, explaining that people say unkind things when they're feeling bad, but it doesn't make it true*.

4. One of your children is quite thin while the others are not, and this leads them to tease the thinner child, especially at meal times.

Try responses like:

"It's unkind to tease people about what they look like – it's good that we look different, or how would we know who's who?"

To the children doing the teasing: "People grow at different rates, as long as you are all active and healthy, that's what counts."

"What do you think about having a family rule that we won't tease each other about our size and how we look? Any ideas of how we could do that?"

SECTION 6: What could I say when…?

5. You find out your child has started following advice they have found on social media about dieting to stay slim or lose weight.

Try responses like:

"I'm interested to hear about what you've found online – can you tell me what it's about?"

"What do you know about how much we can trust what we see online?" *Explain that most of the information is not written by medical professionals and risks harming your health. "*Would you let me help you try and find some websites that we could trust more?"

"A lot of what you see online isn't written by doctors, so we shouldn't really trust it. And I want to keep you safe"

6. Your child sees the feedback letter from the NCMP that says they are overweight, or otherwise is told they are "a bit heavy for their height"

Try responses like:

"People grow at different speeds, and it's hard for all of us to keep active and eat healthily all the time. What do you think we should do differently as a family to keep on track?"

"It looks like you may be a bit heavy for your height, what do you think about that?" *Explain that you want to help you be healthy and that now we know, we can make some healthy changes. Ask your child what changes they would like to try.*

7. My child is always telling me they are hungry, even when we have plenty to eat

"I might not always give you a snack if I know we're going to have lunch or tea soon, just so we get into good habits of enjoying meals and eating healthy things"

"It's important to get the right vitamins and so on when you're growing up, so if you do need snacks sometimes, we just need to make sure they are healthy ones."

"Sometimes people think they're hungry, but they may just be bored, thirsty or not feeling good. Do you think that might be how you feel?"

If you are worried about your child's eating, please see Section 7 for sources of support.

SECTION 6: What could I say when…?

8. Your child is refusing to eat, or is controlling their eating very strictly

Try responses like:

"Are you worried about eating at the moment? How can I help?"

"Can you tell me a bit about why you don't want to eat much at the moment?"

"Sometimes when people are very strict with what they eat they don't get all the vitamins and energy they need to grow properly. I'm worried this might be true for you—what do you think? "

9. Your friend starts telling you about their tough new weight-loss diet, in front of your child

Try responses like:

"I hope you don't mind, but I prefer not to talk about these things in front of my child"

"I'm trying not to talk about dieting in front of the kids. Would you mind if we talked about this some other time when they're not here?"

10. Your child has stopped wanting to do PE or come swimming and says it's because he/she doesn't like people seeing him/her getting changed

Try responses like:

"I'm worried you're missing out on fun things because you don't like people seeing you getting changed. What could I do to help, to make sure you don't miss out?"

"It's normal to be a bit embarrassed getting changed when you are growing quickly and changing a lot. Do you think your friends are worried about this too? What could you do to help each other?"

"Are you worried about how you look? Would you tell me about it in case I can help?"

Any of these examples could be a chance to talk to your child about growth, health, what they eat or the exercise they get – and check in on whether they have any worries about these.

SECTION 7: Links to further advice and information

The links below are to sites that provide reliable information—but please note that as they are provided by other organisations so they may occasionally change or become unavailable.

Tips and ideas for increasing your family's activity and healthy eating

Change4Life - tips on healthy eating, physical activity, weight and mental wellbeing

NHS healthy eating — 8 tips for all the family

NHS activity guidelines for children — information on how much and what types of activity to help your child to do.

How to check whether your child is a healthy weight:-

NHS healthy weight calculator

Being 'media smart'

These sites give advice for parents on how to help their children understand the messages they might see on TV or online.

Common Sense Media

Media Smart

If you have concerns about your child

If you are worried about your child's weight, eating or activity levels, your GP or school nurse should be able to offer guidance and support.

If you are worried that your child may have an eating disorder, BEAT offers information and support.

If you are worried that your child is being bullied or may be bullying others, bullying.co.uk has advice for parents: www.bullying.co.uk/advice-for-parents/

Resources to help support your child's self esteem and body image

Young Minds self-esteem factsheet—advice for parents from the Young Minds organisation

Confident Body, Confident Child—website for an Australian programme to support body confidence in children

Notes and references

References that have informed this guidance

1. Gillison, F., Lorenc, A., Sleddens, E., Williams, S., & Atkinson, L. (2016). Can it be harmful for parents to talk to their child about their weight? A meta-analysis. *Preventive Medicine*, 93, 135-146. https://doi.org/10.1016/j.ypmed.2016.10.010 *(see Appendix 5)*
2. Gillison, F., Grey, E., McConnell, H., & Sebire, S. (2021). The feasibility of using Narrative Messages to Improve Parents' Experience of Learning that a Child is Overweight British Journal of Child Health. *British Journal of Child Health. (see Appendix 6)*
3. McPherson, A. C., Hamilton, J., Kingsnorth, S., Knibbe, T. J., Peters, M., Swift, J. A., Krog, K., Chen, L., Steinberg, A., and Ball, G. D. C. (2017) Communicating with children and families about obesity and weight-related topics: a scoping review of best practices. *Obesity Reviews*, 18: 164– 182. doi: 10.1111/obr.12485.
4. Gillison, F., Cooney, G., Woolhouse, V., Davies, A., Dickens, F., & Marno, P. (2017). Parents' perceptions of reasons for excess weight loss in obese children: a peer researcher approach. *Research Involvement and Engagement*. https://doi.org/10.1186/s40900-017-0072-0
5. Edwards, L. & Reis, S. (2014). A Five-Step Process for Interactive Parent–Adolescent Communication About HIV Prevention: Advice From Parents Living With HIV/AIDS. *Journal of HIV/AIDS & Social Services*, 13:1, 59-78. https://doi.org/10.1080/15381501.2013.775686
6. Randolph, S.D., Coakley, T., Shears, J. and Thorpe, R.J., Jr. (2017), African-American Fathers' Perspectives on Facilitators and Barriers to Father–Son Sexual Health Communication. *Research in Nursing and Health*, 40, 229-236. https://doi.org/10.1002/nur.21789
7. Stone, N., Ingham, R., McGinn, L. & Bengry-Howell, A. (2017) Talking relationships, babies and bodies with young children: the experiences of parents in England. *Sex Education*, 17:5, 588-603. https://doi.org/10.1080/14681811.2017.1332988
8. Hart, E., Chow, C.M. "I just don't want to be fat!": body talk, body dissatisfaction, and eating disorder symptoms in mother–adolescent girl dyads. *Eating and Weight Disord*ers, 25, 1235–1242 (2020). https://doi.org/10.1007/s40519-019-00756-y
9. Kichler, J. C., & Crowther, J. H. (2009). Young Girls' Eating Attitudes and Body Image Dissatisfaction: Associations with Communication and Modeling. *The Journal of Early Adolescence*, *29*(2), 212–232. https://doi.org/10.1177/0272431608320121

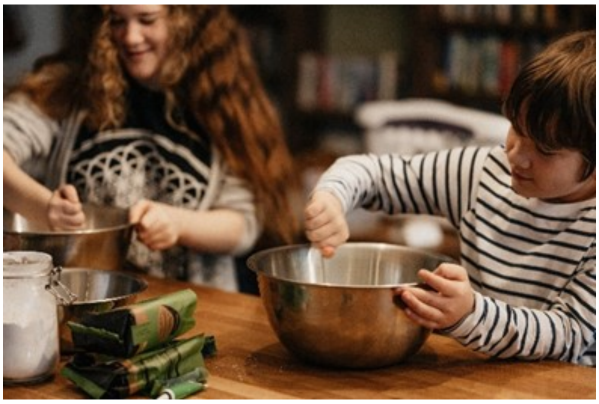

Acknowledgements

We would like to thank all the parents, school nurses, GPs, public health practitioners and academics who provided valuable feedback in developing this guidance. We are also grateful to the children and parents who took part in the research that informed this guidance.

The images used are either owned through shutterstock licence, or downloaded from Unsplash, the Public Health Image Library and the World Obesity organisation images library
